# Exploratory in vitro study of inductive heating–assisted refixation in cemented hip stems

**DOI:** 10.1038/s41598-026-50093-1

**Published:** 2026-05-26

**Authors:** Magnus Reulbach, Patrick Evers, Henning Windhagen, Florian Nürnberger, Eike Jakubowitz

**Affiliations:** 1 Department of Orthopedic Surgery, Laboratory for Biomechanics and Biomaterials (LBB), Hannover Medical School, Anna-von-Borries-Strasse 1-7, 30625 Hannover, Germany; 2 Institut für Werkstoffkunde (Materials Science), Leibniz University Hannover, An der Universität 2, 30823 Garbsen, Germany

**Keywords:** Total hip arthroplasty, Revision surgery, Hip implant loosening, Refixation, Inductive heating, Acoustic emission analysis, Engineering, Materials science

## Abstract

To avoid highly invasive cement extraction during revision of cemented hip stems, we investigated in vitro a concept for refixation of loosened cemented stems using induction heating. The thermoplastic polymeric bone cement is softened by heating the metallic stem above the cement’s glass transition temperature, possibly allowing refixation. In an exploratory study three simplified conical Co28Cr6Mo samples were corundum blasted to simulate the surface roughness of matt cemented stems. Three fixation states were produced by cementing the stem samples within a PMMA cavity: (1) initially implanted stem after cement polymerization; (2) loosened stem retained by taper self-locking; (3) refixated stem. The latter should be achieved by inductive surface heating to 95 °C and applying an axial force of 2 kN. Fixation quality was assessed from relative motions in the stem-cement interface, acoustic emissions during quasistatic torsional loading (7 Nm and 10 Nm), and axial pull-out forces of the stem from the cement mantle. Initial fixation yielded a pull-out force of *F*_PO_ = 1.99 kN ± 0.26 kN, decreasing to 0.84 kN ± 0.38 kN after loosening. After refixation, pull-out forces reached 0.89 kN ± 0.50 kN. However, in one of the three samples, the pull-out force could be restored by refixation. Therefore, the induction-based refixation concept shows potential as a less invasive alternative to conventional revision surgery but requires further validation in more clinically relevant in vitro models.

**Supplementary Information:**

The online version contains supplementary material available at 10.1038/s41598-026-50093-1.

## Introduction

 Aseptic loosening poses a significant risk to the long-term success of total hip arthroplasty (THA)^1^. According to the German Arthroplasty Registry (EPRD), in 2023, aseptic loosening was reported as the leading cause of THA revisions, with the stem being revised in 63 % of revision cases^2^. Aseptic loosening represents a partial or complete loss of the stem fixation in the absence of an infection, due to insufficient stability or particle-induced osteolysis^3^. In cemented THA, this loss of fixation commonly occurs at the stem-cement interface^4^.

The long-term stability of cemented THA depends on the adequate immobilization of the stem in the bone cavity by the PMMA-based bone cement^5^. Clinically, two different stem designs are used to achieve immobilization. These differ fundamentally in their mechanical fixation principles: the “force-closed” and the “composite beam” designs^6^.

With the force-closed principle, a polished surface facilitates controlled subsidence of the stem after debonding, which, in combination with its tapered shape, enables mechanical stabilization through force closure driven by a combination of radial forces and surface friction at the macroscopic level. This design provides excellent long-term stability but is associated with a higher risk of revision due to postoperative periprosthetic fractures^7,8^.

In contrast, the stem of a composite beam design is secured within the bone cement mantle through mechanical retention at a microscopic level due to its matte surface finish. Ideally, this interlocking prevents relative motions in the stem-cement interface. However, complete immobilization is not achievable, leading to an increased risk of revision due to the growth of relative movements in the cement mantle over time^9^. Due to this gradual stem debonding, the roughened surface — actually intended to enhance the fixation — acts like a rasp, accelerating cement mantle wear^9,10^. This process ultimately results in mechanical retention loss and wear particle-induced osteolysis, necessitating revision surgery^9,11–13^.

During a stem revision, the main procedures are removal of both the stem and the cement mantle, along with debridement of the affected soft and hard tissues, before inserting a new stem^14^. Extracting the cement is a complex and time-consuming procedure with the risk of severe complications, including bone perforations and fractures^15,16^. To mitigate these complications, the cement-in-cement revision technique was introduced as an alternative^16^. This technique involves preserving the original bone-faced cement mantle part, into which the new stem is inserted along with new cement^17–19^. The success of this technique depends mainly on the cement mantle quality as contaminations from media like blood, bone marrow, and saline can compromise the new cement-cement interface bond^11,19^. Due to such contaminations, cement-in-cement revisions are reported to result in decreased interface shear strengths of up to 85 %^17,20^.

The problem of the ever-present, progressive loosening of cemented stems should generally be solved to improve long-term stability and thus significantly increase patient safety. In the case of the composite beam design with its matte surface, one solution could be to restore the mechanical retention to its original state. Since this retention is mechanical and the PMMA-based bone cement is a thermoplastic polymer that does not develop adhesive bonding to the metallic stem^10^, refixation could be achieved by briefly heating and softening the cement at the interface. Inductive subcutaneous surface heating of the metallic stem could prevent the need for the extraction of the stem from the bone cement mantle. Simultaneous axial loading of the stem would generate radial forces in the interface again, so that a new microstructural retention can be generated.

In previous in vitro experiments, the effects of physiological conditions on the mechanical and thermal properties of bone cement were investigated^21,22^. Bone cement undergoes a fluid uptake, leading to a higher thermal contact conductance with the metallic stem and a reduction in its glass transition temperature and Vicat softening temperature between 10 °C and 16 °C resulting in values around 90 °C. Nevertheless, Vicat softening measurements showed that even in the non-aged, dry state, thermoplastic PMMA bone cement already exhibits pronounced softening between 90 °C and 95 °C. This suggests that sufficient softening for refixation can be achieved without evaporating the water present at the cement–implant interface. The aim of this exploratory in vitro study is to analyze in a simplified model whether a loosened hip stem can be refixated by pressing it into the previously inductively heated and softened PMMA-based bone cement mantle. Confirmation of the following hypotheses provides initial evidence that this approach could, in principle, be implemented following further adaptation to clinically relevant conditions.

1. Loosening of a cemented hip stem with a matte surface leads to reduced extraction forces.
2. Refixation of the loosened hip stem restores extraction forces.

## Materials and methods

Three different fixation states of the stem within the bone cement mantle were created and analyzed: (1) initial stem stability after implantation, (2) loosened stem with residual force-closed taper self-locking and (3) refixated stem following simultaneous inductive heating and axial loading. Relative micro-motion determination as well as acoustic emission (AE) analysis were employed to assess the fixation state while applying a torque load to the structure. To quantify the resulting bond strength of each state, a universal tensile testing machine was used to load the stem axially for pull-out tests. Consistent with the preliminary nature of the work, no sample-size considerations were applied.

**Fig. 1:**
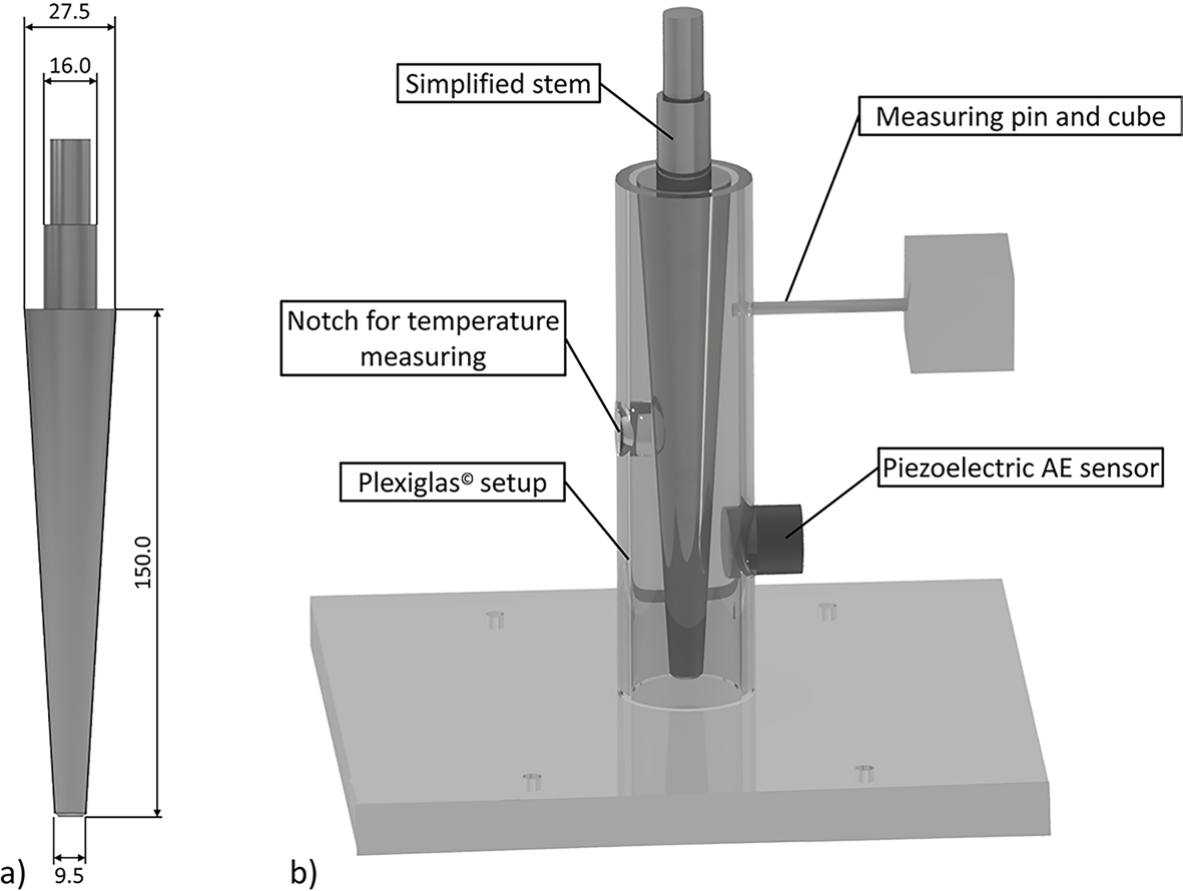
Simplified stem sample (**a**) and sample setup for testing different fixation states (**b**).

In accordance with DIN ISO 5832-12^23^ three simplified stem samples (S1–S3) were turned from Co28Cr6Mo (Fig. 1). They featured a conical, rotationally symmetric taper shape, modelled after one of the most commonly used cemented hip stems in Europe, the Lubinus SP II (Waldemar Link GmbH & Co. KG, Hamburg, Germany)^24^. Subsequently, the fixating sample surfaces were blasted first with corundum Al_2_O_3_ particles (type F60, Hafra GmbH, Assling, Germany) and then with glass beads (100 µm – 200 μm, also Hafra GmbH) in accordance with the manufacturer’s instructions to achieve the typical matte surface of cemented stems.

The femoral cavity was substituted using a PMMA tube (Plexiglas^®^, Röhm GmbH, Darmstadt, Germany) to avoid boundaries of materials with different acoustic impedances in order to reduce acoustic wave reflections during AE analysis. The tube offered an internal diameter of 32 mm and a wall thickness of 4 mm.

Palacos^®^ R bone cement (Heraeus Medical GmbH, Wehrheim, Germany), a radiopaque PMMA-based bone cement, was used to fix the simplified stems inside the PMMA tubes. For this, two standard units of bone cement (2 × 40.2 g polymer powder, 2 × 20 ml liquid monomer) were mixed for 30 s using the Palamix vacuum mixing system (also Heraeus Medical GmbH). The PMMA tube was then retrogradely filled with bone cement, mimicking surgical practice to minimize trapped air in the resulting cement mantle. Subsequently, the stem was inserted into the bone cement using a servohydraulic universal materials testing machine (Type Landmark 100 kN, MTS Systems Corporation, Eden Prairie, USA) to ensure its alignment with the force direction during the following pull-out tests. The process resulted in a bone cement mantle with a minimum thickness of 2.25 mm, adhering to the recommended standards for cemented THA. During implantation, the manufacturer’s recommended processing time of five minutes was not exceeded.

Completion of cement polymerization resulted in the first fixation state, “initial stem stability after implantation”. After the pull-out test, the second state, “loosened but retaining force-closed taper self-locking”, was generated by repeated reinsertion into and extraction out of the cement mantle over ten cycles, where reinsertion was performed using a quasistatic force application of up to 2 kN. This force value corresponds to axial hip joint loadings during level walking^25^ and is intended to simulate the remaining force closure fixation in the cement mantle in accordance with tapered stem shapes. The extraction was carried out with 0.06 mm/s up to a maximum displacement of 2 mm, after which the direction of force was reversed for reinsertion.

For the third fixation state, the refixated stem following simultaneous inductive heating and axial loading, the loosened stem was inductively heated (TruHeat HF 3010, Trumpf, Ditzingen, Germany) at a rate of 0.05 K/s up to a stem surface temperature of 95 °C ± 1 °C, leading to bone cement softening^21^. The temperature was maintained for 5 min before the stem was pressed back into the cement mantle at a constant speed of 0.1 mm/s until a force of 2 kN was reached. Through an iterative process, a waiting time of five minutes was found to be optimal for allowing sufficient heat conduction through the cement mantle. The insertion force corresponds to the 2 kN applied to generate mechanical interlocking in the loosened state, thereby providing standardization and ensuring comparability between the fixation conditions. Once the force was reached, it was held constant to enable refixation, while the system passively cooled to ambient temperature over approximately 30 min. The surface temperature of the stem was monitored using a pyrometer (Type CTlaser LT, Optris GmbH, Berlin, Germany), aligned to the stem surface through a notch in the PMMA tube and the bone cement mantle (Fig.1). The notch was positioned at the center of the stem, 75 mm from the proximal edge of the cemented stem.

The Helmholtz inductor consisted of two opposing coils, each with two turns and a spacing between coils of 70 mm. The coils were adapted to the conical shape of the stem. In the medio-lateral direction, each coil was 15 mm wider than the stem, while in the axial direction the coil dimensions exceeded those of the stem by 10 mm, aiming to produce a homogenous magnetic field across the stem geometry. In the axial direction, the maximum coil dimensions were limited by the clamping device of the materials testing machine. The stem was positioned centrally between the two coils. It was aligned such that the notch for measuring the stem surface temperature was oriented perpendicular to the cross-sectional plane of the Helmholtz coils, where the highest magnetic flux density is expected.

### Torsional force application

**Fig. 2:**
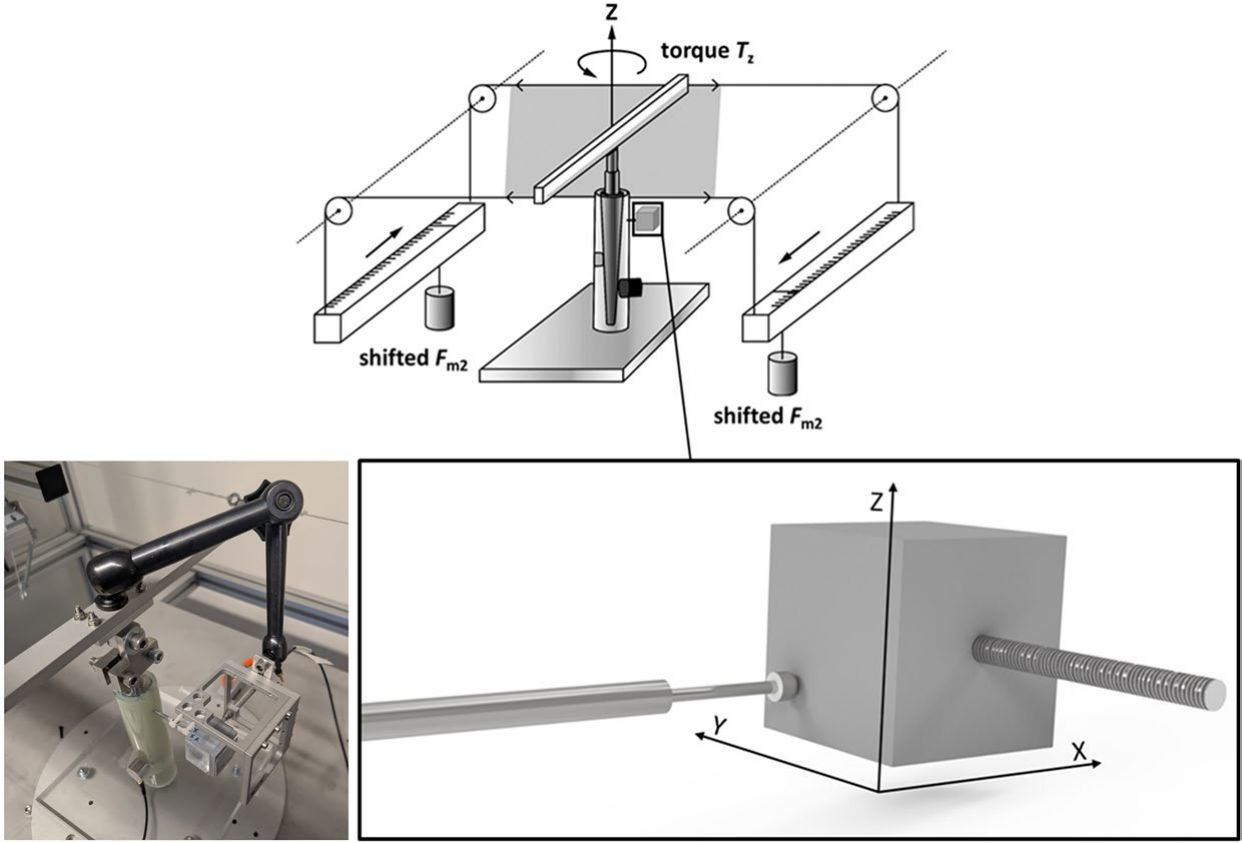
Setup for torsional torque application and linearly variable differential transducers for relative motion measurement.

Since the largest relative motions are expected around the longitudinal axis Z of the stem due to its rotationally symmetric geometry, an axial torque *T*_Z_ was applied to the stem via a lever arm. Via elastic ropes, the lever arm was connected to two linear motor units, which each drive weights in a reciprocal manner (Fig. 2). This setup ensures a continuous increase of *T*_Z_, which was applied in two stages for each fixation state. The first stage involved a maximum value of *T*_Z_ = ± 7 Nm, whereas the second ranged up to *T*_Z_ = ± 10 Nm. Since acoustic emissions due to the debonding processes are non-repetitive and irreversible events, a total of *n* = 25 loading cycles was performed for each stage and fixation state. Linear motor units were controlled using LabVIEW (version 2022 Q3; National Instruments, USA; ni.com). All tests were conducted at the same room temperature (24 °C ± 1 °C) and humidity (40 % ± 10 %).

A measuring frame equipped with a differential transducer (Type P2010, Mahr GmbH, Göttingen, Germany) with a resolution of 0.1 μm was attached to the stem. This transducer determined the stem’s relative rotational motion with respect to a cube attached to the cement mantle with a standardized distance via a metal pin (Fig. 2). The quasistatic torque application allowed for a relative motion capturing subdivided into 40 steps (∆*T*_Z_ = 0.175 Nm for *T*_Z_ = ± 7 Nm; and ∆*T*_Z_ = 0.250 Nm for *T*_Z_ = ± 10 Nm). The transducer data were acquired using LabVIEW (version 2022 Q3; National Instruments, USA; ni.com).

For simultaneous AE detection, a passive piezoelectric AE sensor (VS45 H, Vallen Systeme GmbH, Wolfratshausen, Germany) with a frequency range of *f* = 20 kHz – 450 kHz was placed onto the bone cement mantle (Fig. 1). The sensor has a sensitivity of -62 dB re 1 V/µbar, with a peak frequency of 280 kHz, corresponding to an output voltage of approximately 2.5 mV at an acoustic pressure of 1 µbar. It is operated with an AMSY6 AE acquisition system and an AEP3N preamplifier with an amplification of *T* = 49 dB (both also from Vallen Systeme GmbH). Data acquisition was performed at a sampling rate of *f*_S_ = 5 MHz using the corresponding acquisition software (version R2021.1122.1, Vallen Systeme GmbH, Germany, vallen.de).

After cyclic torque application, pull-out tests were performed using the MTS Landmark 100 kN universal testing machine. Results are presented as mean ± standard deviation (s.d.) based on three specimens per fixation condition.

The processing and visualization of the pull-out tests and the determination of rotational stability as well as AE signal processing and characterization were performed using custom Python code. All analyses were conducted in Python (v3.10; Python Software Foundation, python.org). The implementation relied on the following libraries: NumPy (v1.24.4, numpy.org), Pandas (v1.4.3, pandas.pydata.org), SciPy (v1.8.1, scipy.org), Matplotlib (v3.5.2, matplotlib.org), Seaborn (v0.12.2, seaborn.pydata.org), and vallenae (v0.8.0, github.com/vallen-systems/vallenae).

The AE signals were filtered using a fifth-order Butterworth bandpass filter (20 kHz – 450 kHz) to restrict the signal to the AE sensor’s operating range. AE events (hits) were distinguished from noise by their higher amplitude. A defined voltage threshold, which must be overcome for hit detection, was set (*U*_Thr_ = 0.08 mV). Three parameters were defined, for which hits were considered valid after the AE signal exceeded the predefined threshold: hit definition time (*t*_HDT_ = 0.10 ms), hit lockout time (*t*_LOT_ = 0.06 ms), and peak definition time (*t*_PDT_ = 1.00 ms).

The hit definition time specifies the maximum time between threshold crossings. When the hit definition time is exceeded without threshold crossing, a hit is defined as completed. The hit lockout time refers to the minimum time interval required between the detection of two consecutive hits. The peak definition time represents the time that can pass after the threshold has been exceeded until the maximum amplitude of the hit is reached^26^.

The energy of each individual hit was calculated by summing the squared voltage samples and is expressed in arbitrary energy units (eu). To estimate the energy released during loosening events, the cumulative energy of all detected AE hits was determined for each loading increment and aligned with the corresponding motion measurement increment. It can be assumed that the greater the mechanical energy accumulated at the stem-cement interface before abrupt relative movement occurs, the higher the energy released in the corresponding AE events. For comparing fixation states, the AE signal energy of the entire measurement session was summed over all 25 load cycles. Additionally, the summed energy was normalized to the number of detected AE hits.

### Contact area determination

Engineers Blue was used to qualitatively assess the contact area after refixation. After the pull-out tests, the inner surface of the cement mantle — representing the contact interface between cement and stem — was uniformly coated with the blue oil paint. The hip stem was then pressed axially into the cement mantle with a force of 2 kN, replicating the interface contact pressure of the two fixation states, and subsequently pulled out. The paint adhered to the stem at contact regions. To document the contact pattern, four photographs were taken, rotating the stem by 90° between each image.

## Results

### Pull-out forces

**Fig. 3:**
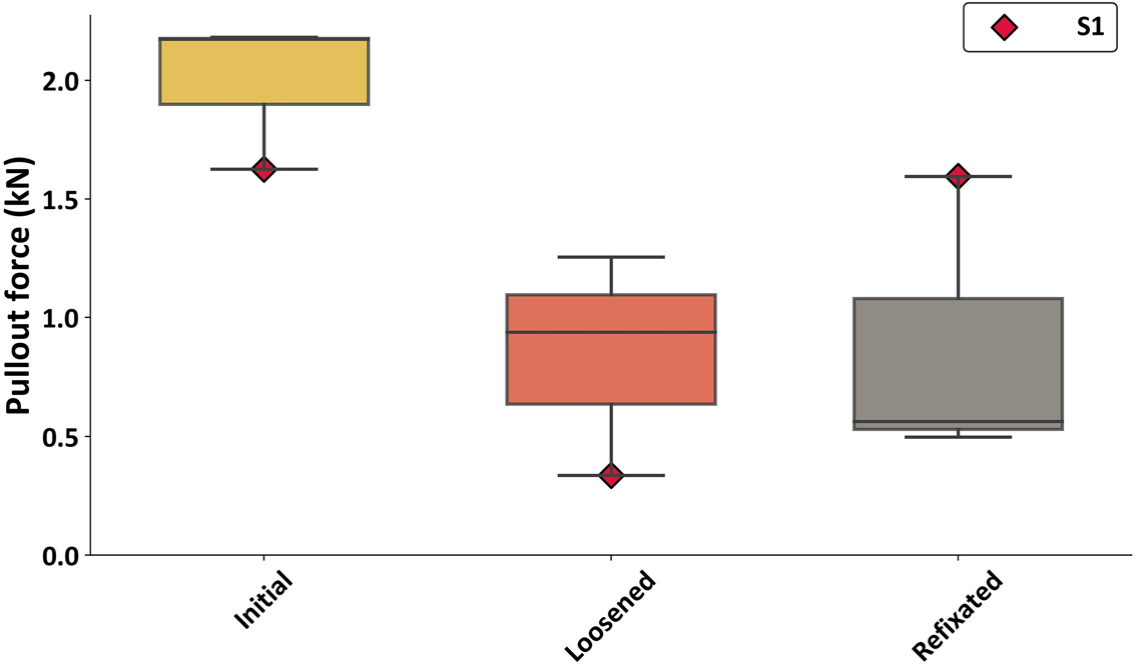
Pull-out forces F_PO_ for initial fixated, loosened and refixated fixation states. Forces for S1 are highlighted in red.

The initial fixation state yielded the highest pull-out forces, with an average of *F*_PO_ = 1.99 kN ± 0.26 kN. The loosened state resulted in *F*_PO_ = 0.84 kN ± 0.38 kN. In contrast, the mean pull-out force for the refixated state (*F*_PO_ = 0.89 kN ± 0.50 kN) did not change compared to the loosened one (Fig. 3). Individual analysis of S1 revealed distinctly different results compared to the other samples. In this case, the pull-out force decreased from *F*_PO_ = 1.63 kN (initial fixation state) to *F*_PO_ = 0.33 kN after loosening. However, refixation following heat treatment restored the pull-out force to *F*_PO_ = 1.60 kN. In contrast, the refixation process resulted in a reduction of pull-out forces compared to the loosened state in samples S2 and S3.

### Torsional relative motion of the stem and associated acoustic emissions

With a torque load of ± 7 Nm, no relevant differences in the mean relative motion in the stem-bone interface between the fixation states were present (Table 1). When the load was increased to ± 10 Nm, the relative motion was approximately twice as high.

**Table 1:** Motion range, cumulative energy, number of hits and energy/hit for the states initial fixated, loosened and refixated stem with 7 Nm and 10 Nm maximal torque load.

Axial torque T_z_	Test Condition	Sample	Motion range in mm	Summed energy in eu	Number of hits	Energy/Hit in eu/hit
**7 Nm**	**Initial**	S1	0.065	8.0 × 10^− 1^	7	1.1 × 10^− 1^
		S2	0.063	6.1 × 10^+ 2^	80	7.6 × 10^+ 0^
		S3	0.079	9.3 × 10^+ 3^	29	3.2 × 10^+ 2^
		**Mean**	**0.068**	**3.3 × 10** ^**+ 3**^	**39**	**1.1 × 10** ^**+ 2**^
		*SD*	0.007	4.3 × 10^+ 3^	31	1.5 × 10^+ 2^
	**Loosened**	S1	0.126	2.0 × 10^+ 2^	68	2.9 × 10^+ 0^
		S2	0.035	5.3 × 10^− 1^	4	1.3 × 10^− 1^
		S3	0.075	7.9 × 10^+ 4^	164	4.8 × 10^+ 2^
		**Mean**	**0.079**	**2.7 × 10** ^**+ 4**^	**79**	**1.6 × 10** ^**+ 2**^
		*SD*	0.037	3.7 × 10^+ 4^	66	2.3 × 10^+ 2^
	**Refixated**	S1	0.070	1.1 × 10^+ 5^	91	1.2 × 10^+ 3^
		S2	0.063	3.3 × 10^+ 6^	148	2.2 × 10^+ 4^
		S3	–	–	–	–
		**Mean**	**0.067**	**1.7 × 10** ^**+ 6**^	**120**	**1.2 × 10** ^**+ 4**^
		*SD*	0.004	1.6 × 10^+ 6^	29	1.1 × 10^+ 4^
**10 Nm**	**Initial**	S1	0.142	4.8 × 10^+ 4^	107	4.5 × 10^+ 2^
		S2	0.149	1.4 × 10^+ 6^	495	2.9 × 10^+ 3^
		S3	0.149	4.5 × 10^+ 5^	284	1.6 × 10^+ 3^
		**Mean**	**0.147**	**6.4 × 10** ^**+ 5**^	**295**	**1.6 × 10** ^**+ 3**^
		*SD*	0.004	5.7 × 10^+ 5^	159	9.9 × 10^+ 2^
	**Loosened**	S1	–	–	–	–
		S2	0.159	1.5 × 10^+ 6^	501	3.0 × 10^+ 3^
		S3	0.180	5.2 × 10^+ 5^	476	1.1 × 10^+ 3^
		**Mean**	**0.170**	**1.0 × 10** ^**+ 6**^	**489**	**2.0 × 10** ^**+ 3**^
		*SD*	0.011	4.9 × 10^+ 5^	13	9.5 × 10^+ 2^
	**Refixated**	S1	0.151	1.2 × 10^+ 6^	324	3.6 × 10^+ 3^
		S2	–	–	–	–
		S3	–	–	–	–
		**Mean**	**0.151**	**1.2 × 10** ^**+ 6**^	**324**	3.6 × 10^+ 3^
		*SD*	–	*–*	–	*–*

S1 showed a larger offset during one cycle in the loosened state, resulting in a higher mean relative motion. However, the 10 Nm loading in S1 in the loosened state led to a complete implant-cement interface failure, terminating the measurement (Fig. 5). In the refixated state, the same interface failure occurred for S2, whereas for S3, this even occurred before the full achievement of 7 Nm (Fig. 6).

**Fig. 4:**
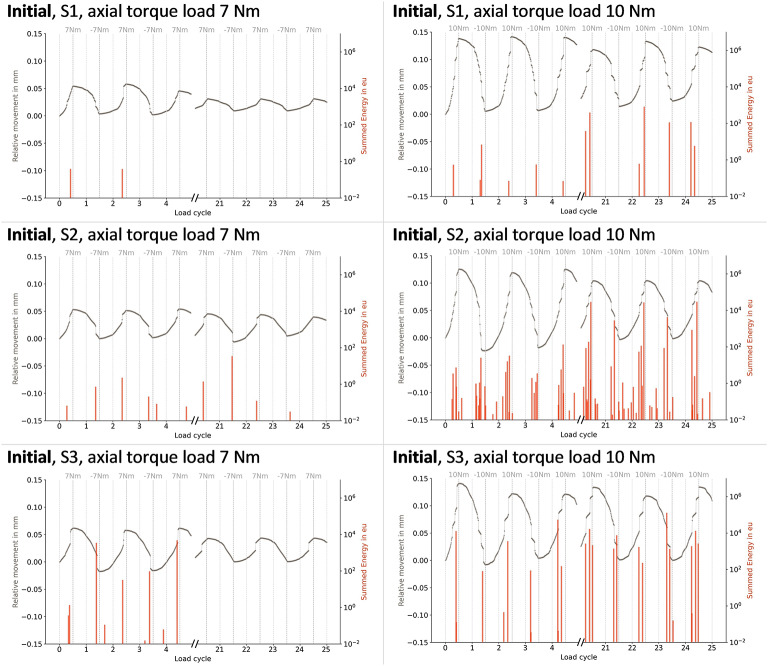
Relative motion and summed acoustic energy during cyclic torsional loads of up to ± 7 Nm and ± 10 Nm in the **initial** fixation state, showing the first and last five load cycles for S1–S3.

**Fig. 5:**
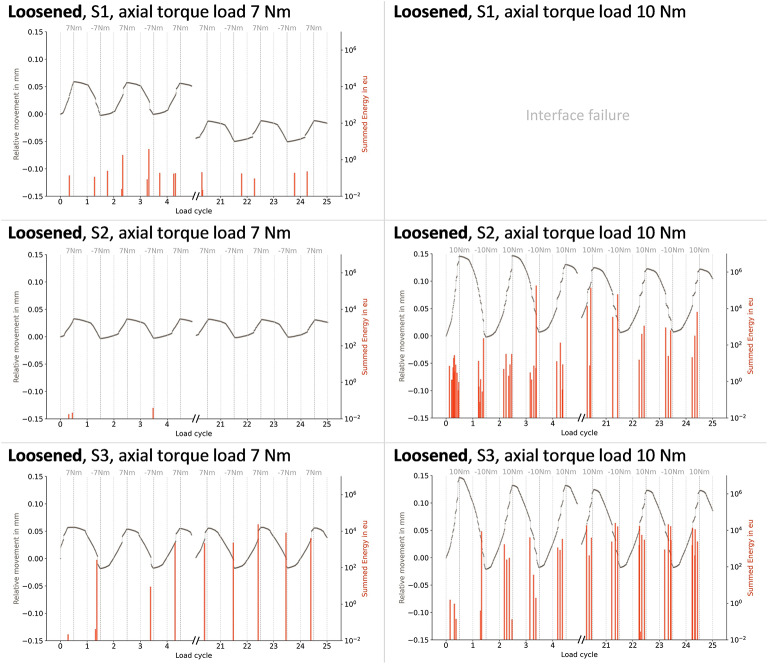
Relative motion and summed acoustic energy during cyclic torsional loads of up to ± 7 Nm and ± 10 Nm in the **loosened** fixation state, showing the first and last five load cycles for each sample (S1, S2, and S3). Empty fields indicate mechanical interface failure during measurement, causing measurement termination.

Across all fixation states, AE signals coincided with the onset of abrupt relative motions. Increasing the maximum torque to 10 Nm resulted in a higher number of AE signals and greater cumulative energy during larger abrupt motions (Fig. 4).

**Fig. 6:**
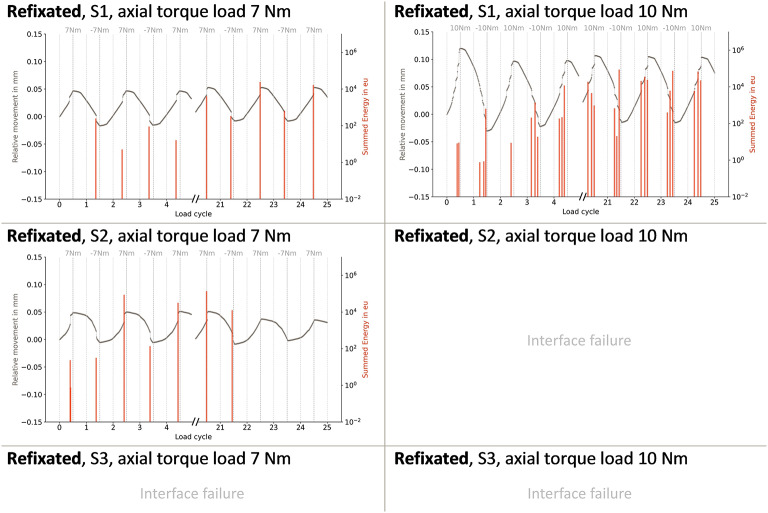
Relative motion and summed acoustic energy during cyclic torsional loads of up to ± 7 Nm and ± 10 Nm in the **refixated** fixation state, showing the first and last five load cycles for each sample (S1, S2, and S3). Empty fields indicate mechanical interface failure during measurement, causing measurement termination.

In general, during the 7 Nm torque application, the number of AE signals always decreased over the course of 25 cycles in the initial fixation state. In the loosened state, this number increased, whereas the energy per hit remained unchanged. Both the number and the mean energy per hit increased during the refixated state. When the load was raised to 10 Nm, differences between fixation states became less pronounced, both in terms of the number of hits and the energy per hit.

During the initial fixation state of S1, almost no AE signals at 7 Nm were present (Fig. 4). In contrast, S2 showed fewer relative motions during the loosened fixation state compared to the initial state (Fig. 5). This was accompanied by considerably fewer AE hits with additionally lower energy per hit.

### Contact area

**Fig. 7:**
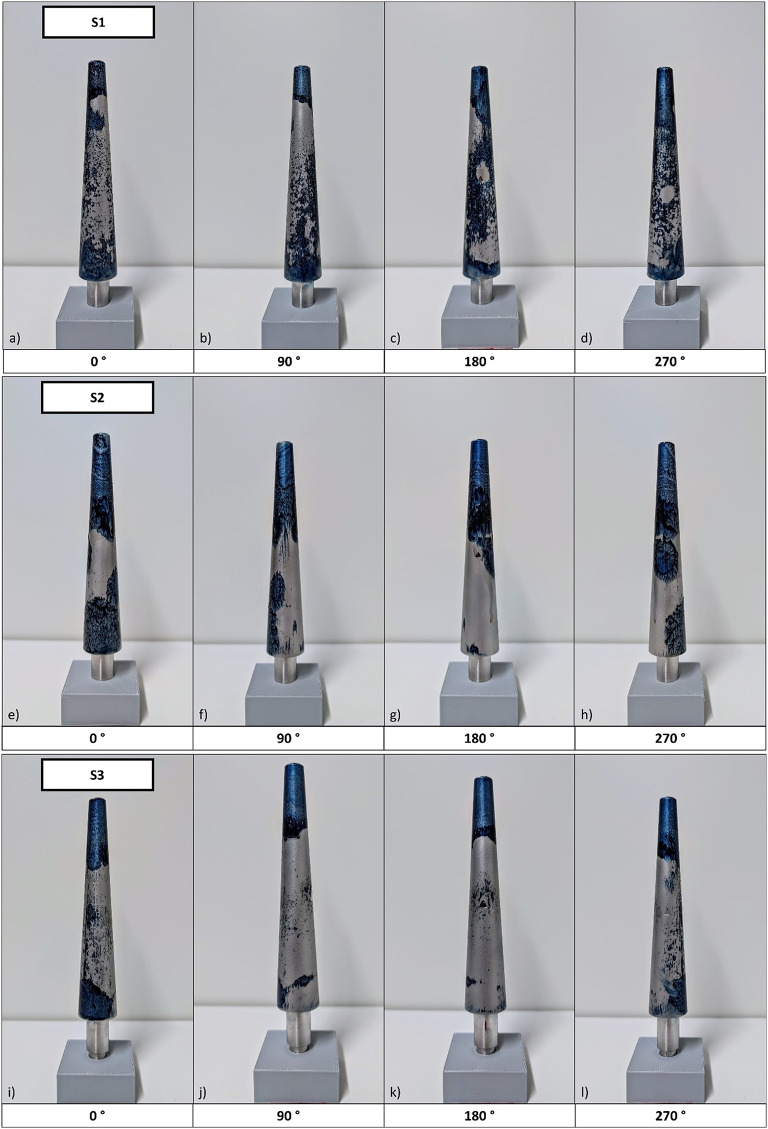
Contact surface analysis using engineer’s blue of samples S1—S3.

After refixation, gaps in the contact surface are visible in S2 and S3, particularly in the proximal and central areas of the stem. For these samples, only the distal part of the stem shows a circular closed contact area. S1 has an enclosed contact area in both the proximal and distal stem parts, although smaller gaps are still present in the central stem area (Fig. 7).

## Discussion

The highest pull-out forces were measured for the initial fixation state. However, when the pull-out forces normalized to the sample surface are compared with literature values regarding pure shear bond strength, they are almost 95 % – 98 % lower^10,27^. This discrepancy is likely linked to the conical shape of the present simplified stem samples since the axial tensile load is actually divided into interface shear and tensile loads, while the mechanical retention of the matte surface is less able to resist the tensile component of forces. Zelle et al. (2011) addressed this divided loading situation and found comparable interface strengths^28^.

As anticipated, the simulated stem loosening state resulting from a repeated pull-out and reinsertion approach reduced the bond strength by approximately 60 % after only ten load cycles. Pressing the stem with its tapered shape into the cement mantle resulted in a force-closed, self-locking mechanism between the stem and the bone cement mantle on a macroscopic scale, similar to the fixation mechanism of a cemented, force-closed stem type with polished surfaces^29^. The observed reduction in pull-out forces suggests that the rough surface did not interlock or only slightly interlocked with the bone cement mantle at the microscopic level when pressed into it at room temperature.

In one of the three samples the refixation method increased the bond strength by 374 % compared to the loosened state. This restored the pull-out force in this sample to nearly the same level as the initial fixation state after polymerization. These preliminary findings in the simplified model, suggest that heating may facilitate both the macroscopic interlocking of the stem and the microscopic refixation. The AE analysis supports this assumption. In the loosened state of S1, only a few AE events with low energy were present, at a maximum torque of 7 Nm, whereas in the refixated state, the events are clearly stronger. To further explore this observation, a post-hoc paired t-test was conducted on the total AE energy across the 25 load cycles, revealing a significant difference between the loosened and refixated state at a maximum torque load of 7 Nm (*p* < 0.05). The statistical evaluation of the AE results is limited to this pairwise comparison, as a holistic assessment of the AE results is not possible due to mechanical interface failure during some AE measurements. This occurred particularly in the refixated state of sample S3, meaning that AE results are not available for all measurement conditions. This higher AE energy in the refixated state suggests that a microscopic interlocking can once again withstand increased stresses. However, when the applied torque likely exceeds the strength of this interlocking, a new detachment process is initiated. The torque stores mechanical energy at the interface, which is then released through sudden relative movements, resulting in clearly detectable AE signals.

In accordance with this mechanism, unexpectedly strong AE signals were observed at a torque load of 7 Nm in the refixated state. This indicates that the microscopic interlocking has not yet been fully re-established. It can be assumed that increased loading of the rotationally symmetric, simplified stem caused the interlocking to fail again at the microscopic level, generating AE signals with higher energy. Moreover, pull-out tests indicate that, even at the current stage of method development, the refixation shows promising results in S1.

In S1, a level of fixation comparable to that of existing, more invasive revision techniques could be achieved, although this was demonstrated under highly simplified in vitro conditions. Currently, the only revision method that allows the initial cement mantle to remain in the bone in cases of implant loosening is the cement-in-cement technique. However, this approach is considerably more invasive than the method investigated in this study and also reduces the shear bond strength at the cement-cement interface to 85 % of its initial state^20^.

With the heating strategy employed in this study, which resulted in successful refixation in one of the three samples (S1), the temperatures during inductive heating remained within the range of those achieved during the initial polymerization of the bone cement or during mechanical cement removal in established revision procedures^30,31^. In addition, García-Galan et al. 2024^32^ demonstrated in a rabbit in vivo model that short-term inductive heating of Ti6Al4V implants, implanted in the femur, to 70 °C did not result in an increased incidence of necrosis in the periprosthetic bone tissue, despite a direct interface between the bone and the implant. This suggests that the method does not substantially exceed established temperature levels and therefore presumably does not cause excessive thermal damage to the surrounding tissue, provided that the heating strategy can be validated under clinically relevant conditions. Nevertheless, in future studies after finalizing the heating strategies for clinically relevant stem and cement geometries, the effect on the periprosthetic tissue has to be systematically validated. However, heating strategies adapted to the stem geometry and site of loosening promise a decrease in total heat applied, resulting in a further reduction of thermally induced damage.

Since the refixation was only successful for the sample S1 in the simplified in vitro setup, the current standardized refixation protocol should be optimized. Based on preliminary investigations into suitable heating strategies and follow-up analyses of all samples, several potential factors contributing to the inconsistencies of refixation were identified. In pre-tests conducted during the development of the current inductive heating strategy, the applied force of 2 kN was not maintained constantly during the cooling phase due to the absence of active force control, in contrast to the strategy presented in the present study. It had been initially assumed that the rigidity of the test setup would ensure a constant contact force. However, this was not the case, resulting in a complete detachment of the stem from the cement mantle. This detachment was likely caused by thermal shrinkage of the bone cement, which led to separation at the implant-cement interface while the stem remained fixed in the tensile-compression testing machine^33^. Even with force control, detachment may still occur if the conical cement mantle does not perfectly conform to the shape of the stem. In such cases, shrinkage during cooling could lead to localized separation at points where contact pressure is lower.

Another weakness in the present refixation protocol is the use of a simplified PMMA bone model. Prolonged heating of the metallic component is assumed to have caused excessive softening of the entire bone cement mantle, including the surrounding PMMA bone model, which features a nearly identical softening temperature. Inconsistent contact during stem insertion, limited to the proximal part of the cement mantel, may have led to localized bulging of the cement mantle and the PMMA tube, as structural support was reduced due to the potential softening of the bone model itself. This effect likely reduced the contact surface for refixation, limiting fixation primarily to the most proximal and distal parts of the conical stem while leaving the central section insufficiently secured. These assumptions were supported by the examination of the contact area between the bone cement and the stem visualized using engineer’s blue. Samples S2 and S3, in which refixation had failed, exhibited a clearly reduced contact area compared to S1. As expected, in S2 and S3, the remaining contact was primarily located in the proximal region of the stems (Fig. 7g) and k)). Additionally, contact in these samples did not occur circumferentially in the proximal part. This suggests insufficient transverse force compensation during insertion, potentially caused by the rigid fixation of the components. Future studies should allow relative transverse mobility between stem and cement mantle to enable proper force equilibration, whereas the femur should be substituted by a non-thermoplastic material comparable to bone. The current heating strategy, which slowly increases the temperature of the bone cement mantle to 95 °C, might not only influence the mechanical success of refixation with the simplified in vitro PMMA bone model but could also lead to necrotic effects in the surrounding tissue in vivo due to excessive heat exposure.

A two-stage heating process could address these challenges. Given that PMMA bone cement starts to soften at 60 °C^21^, this approach could involve gradually heating a broad layer of the cement mantle to a lower initial temperature, allowing adaptation to the stem’s macroscopic shape without leading to tissue damage. Subsequently, briefly applying more intense heating to a cement layer several micrometers thick — within the range of the stem’s surface roughness — could further soften the interface to a level comparable to that of unpolymerized fresh cement. This would allow the bone cement to flow into the cavities of the surface roughness, promoting interlocking between the matte stem surface and the bone cement. During this targeted boundary layer heating, the slow heat conduction and the continuous dissipation of heat through blood perfusion in the surrounding tissue are assumed to keep the contact area between the bone cement and the bone tissue below temperatures that could critically affect human tissue. However, the induction generator used in this study was unable to generate sufficient power to achieve the required stem temperature within a short time. Consequently, the adapted heating strategy could result in more distinct differences between the various fixation states and more reliable refixation.

In older, widely referenced, literature loosening in cemented hip stems is defined as a radiolucent gap of 1mm – 2 mm^34^. However, more recent studies indicate that loosening begins with debonding at the cement stem interface, characterized by radiolucent gaps of just 0.1 mm, although this cannot be reliably detected on a conventional radiograph^35^. A macroscopic mismatch in the loosening gap on the scale of tenths of a millimeter at early stages could likely be compensated for by heat-induced softening of the bone cement mantle. However, based on the current findings, the method may reach its limits in cases of larger, potentially localized defects in the bone cement mantle.

AE analysis has proven to be an effective tool for assessing the fixation state of an implant at this proof-of-concept stage using simplified sample geometries and homogeneous material without soft tissue. Notably, AE analysis was particularly effective in detecting abrupt relative motions between the simplified stem and the cement mantle. High-energy AE signals were observed in instances of stronger relative motions. AE signals with notably higher energy were also detected in the case of the stronger relative motions resulting from the larger applied torque of 10 Nm. Considering the studies by Bergman et al.. (2016)^25^, which reported torque loads exceeding 50 Nm on the stem during activities of daily living, it can be inferred that even stronger AE signals would occur in vivo. This effect might even be more pronounced if superimposed axial loading is involved. The increased signal strength could facilitate the differentiation of AE signals related to implant debonding processes from interference signals generated by other structures within the human body during in vivo use. The promising results of AE in distinguishing between different fixation states in the simplified model indicate that AE technology may also be suitable for diagnosing implant loosening, potentially enabling detection earlier than conventional X-ray diagnostics. In addition, the use of acoustic emission for loosening diagnosis in hip endoprostheses is currently an active area of research^26^.

Due to the exploratory nature of this study, further limitations exist regarding the transferability of the results to the in vivo situation. The primary limitation of assessing rotational stability is the rotational symmetry of the simplified stem, which does not exist in real implants. Clinically employed implants feature specifically designed geometries to enhance rotational stability^29^. Such a geometry would result in much lower relative motion under rotational loading conditions and require higher torque loads to promote the debonding of matte implant surfaces. This, in turn, would presumably lead to different acoustic emission patterns and a higher resistance to acoustic emission when subjected to physiological torques. Furthermore, the mechanical evaluations cannot be directly transferred to the in vivo situation and therefore require validation in human bone specimens in a subsequent in vitro step, as the cement mantle was anchored in a PMMA cavity, which differs from bone in both its thermal and mechanical properties. Nevertheless, using a simplified geometry was essential for standardizing implantation procedures and reducing systemic differences between experimental conditions caused by the heterogeneity of donor femurs. This exploratory approach enabled the controlled testing of the proposed method for refixation of loosened cemented hip stems under reliable conditions.

## Conclusion

Despite its limitations, this exploratory study provides valuable insights into the thermal refixation of cemented hip stems for further method development. In one sample of a simplified hip stem, refixation was achieved by pressing it into the bone cement after inductive heating of the stem resulting in increased bond strength compared to the loosened condition, reaching pull-out forces comparable to the initial fixation state. To achieve these promising results more consistently, the heating protocols used in this preliminary study have to be continuously refined and adapted. With an optimized heating strategy, it might be possible to reliably restore the original fixation state and achieve a comparable level of stability. If these optimized strategies show success, they have to be mechanically validated in a non-thermoplastic bone model. Additionally, AE analysis showed promising results for assessing the state of fixation, by detecting potential debonding processes where energy is abruptly released. This study lays the methodological foundation for the development of a procedure for the thermal refixation of cemented hip stems, potentially avoiding highly invasive revision surgeries for aseptically loosened cemented hip stems, reducing the associated risk of complications for patients.

## Electronic Supplementary Material

Below is the link to the electronic supplementary material.

Supplementary Material 1

## Data Availability

Data will be made available on request.
