# Supplementary figures and images for: Exploratory in vitro study of inductive heating–assisted refixation in cemented hip stems
Source: Sci Rep. 2026 May 26;16:16278. doi: 10.1038/s41598-026-50093-1 (PMC13212937; doi:10.1038/s41598-026-50093-1)

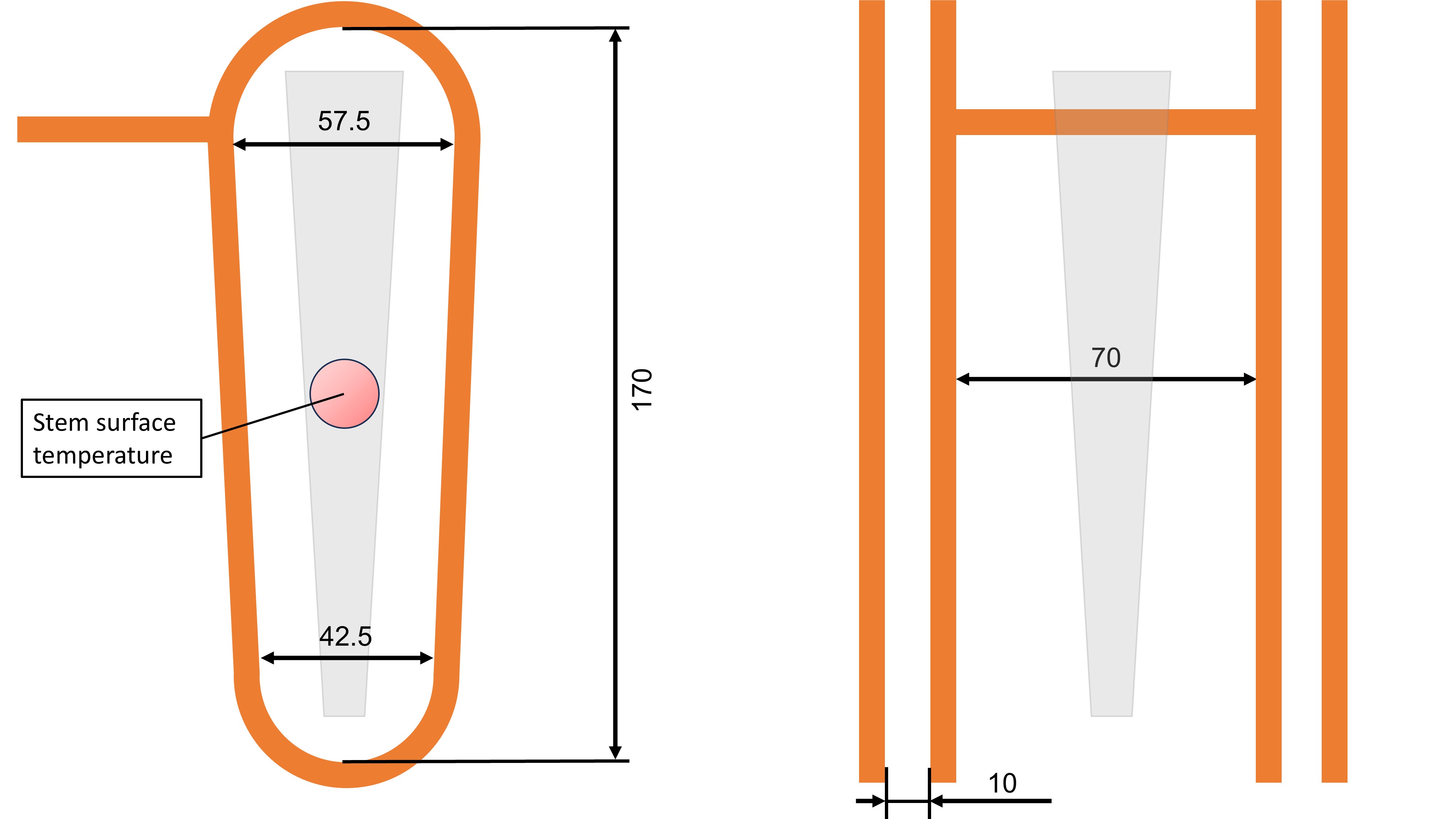

Supplement: Supplementary file 1 — Supplementary Material 1 [file 41598_2026_50093_MOESM1_ESM.jpg]
